# Genetic analysis of the postsynaptic transmembrane X-linked neuroligin 3 gene in autism

**DOI:** 10.5808/gi.21029

**Published:** 2021-12-31

**Authors:** Rajat Hegde, Smita Hegde, Suyamindra S. Kulkarni, Aditya Pandurangi, Pramod B. Gai, Kusal K. Das

**Affiliations:** 1 Laboratory of Vascular Physiology and Medicine, Department of Physiology, Shri B.M Patil Medical College, Hospital and Research Centre, BLDE (Deemed to be University), Vijayapura, Karnataka 586101, India; 2 Karnataka Institute for DNA Research (KIDNAR), Dharwad, Karnataka 580003, India; 3 Human Genetics Laboratory, Department. of Anatomy, Shri B.M Patil Medical College, Hospital and Research Centre, BLDE (Deemed to be University), Vijayapura, Karnataka 586101, India; 4 Department of Psychiatry, Dharwad Institute of Mental Health and Neurosciences, Dharwad, Karnataka 580008, India

**Keywords:** autism, India, missense variant, neuroligin 3

## Abstract

Autism is a complex neurodevelopmental disorder, the prevalence of which has increased drastically in India in recent years. Neuroligin is a type I transmembrane protein that plays a crucial role in synaptogenesis. Alterations in synaptic genes are most commonly implicated in autism and other cognitive disorders. The present study investigated the neuroligin 3 gene in the Indian autistic population by sequencing and in silico pathogenicity prediction of molecular changes. In total, 108 clinically described individuals with autism were included from the North Karnataka region of India, along with 150 age-, sex-, and ethnicity-matched healthy controls. Genomic DNA was extracted from peripheral blood, and exonic regions were sequenced. The functional and structural effects of variants of the neuroligin 3 protein were predicted. One coding sequence variant (a missense variant) and four non-coding variants (two 5'-untranslated region [UTR] variants and two 3'-UTR variants) were recorded. The novel missense variant was found in 25% of the autistic population. The C/C genotype of c.551T>C was significantly more common in autistic children than in controls (p = 0.001), and a significantly increased risk of autism (24.7-fold) was associated with this genotype (p = 0.001). The missense variant showed pathogenic effects and high evolutionary conservation over the functions of the neuroligin 3 protein. In the present study, we reported a novel missense variant, V184A, which causes abnormal neuroligin 3 and was found with high frequency in the Indian autistic population. Therefore, neuroligin is a candidate gene for future molecular investigations and functional analysis in the Indian autistic population.

## Introduction

Autism (MIM 209850) is a complex neurodevelopmental disorder that is characterised by impaired verbal and nonverbal communication and social interaction, accompanied by restricted and stereotyped behavior [1,2]. The American child psychiatrist Leo Kanner was the first to clearly define the condition and to use the term “autism” [3,4]. The aetiology of autism is largely unknown, but many studies have shown that genetic factors play a major role, along with environmental factors. The genetic architecture of autism is complex. Autism shows diverse forms of genetic variation, differing in frequency (i.e., very rare, rare, and common variations), the pattern of inheritance (i.e., autosomal, X-linked, and *de novo* variations), the type of variation (i.e., structural—including aneuploidy, copy number variations, indel mutations, and single-nucleotide variations), and mode of action (additive, recessive, dominant, and hemizygous) [5,6]. The causes of autism may be heritable, de novo, or both. Some family and twin studies have shown that autism is highly heritable. The concordance rate of autism is roughly 45% for monozygotic twins and 16% for dizygotic twins [7,8]. The co-occurrence rate of autism in siblings is approximately 45 times greater than in the general population. The male to female ratio is 3‒4:1 [4,9]. The reason for the male predominance is still unknown.

Various studies from Asia, Europe, and North America have identified the average prevalence of autism spectrum disorder (ASD) to be 1% to 2% [10]. In India, the prevalence of autism has increased drastically over time, and the true reason for the cause of this change remains unclear. In a systematic review of four studies, one study included both rural and urban populations, while the remaining three included only urban populations. The study from the rural cohort showed a pooled prevalence of 0.11% (95% confidence interval [CI], 0.01% to 0.20%) in children of 1‒18 years. A study conducted in the urban cohort showed a pooled prevalence of 0.09% (95% CI, 0.02% to 0.16%) in children aged 0‒15 years [11]. A study conducted in 2017 amongst children from rural, urban, and tribal populations of children aged 1‒10 years showed a prevalence of 0.15% (95% CI, 0.15% to 0.25%) [10].

Autism has diverse pathophysiological mechanisms, of which synaptic cell adhesion and associated molecules are currently amongst the most studied. Neuroligin is a type I transmembrane protein whose extracellular segments contain a globular domain homologous to acetylcholine esterase and a stalk rich in O-linked carbohydrates [12]. In the human genome, five genes have been identified that code for neuroligin: *NLGN1* (3q26), *NLGN2* (17p13), *NLGN3* (Xq13), *NLGN4X* (Xp22.3), and *NLGN4Y* (Yq11.2). The protein products of NLGN1 are located at excitatory synapses, those of *NLGN2* are found at inhibitory synapses, those of *NLGN3* are at both excitatory and inhibitory synapses, and those of *NLGN4X* are found at excitatory synapses. *NLGN4X* and *NLGN4Y* have almost identical sequences, and are therefore assumed to have the same function [13,14]. In the present study, we recorded the prevalence of autism in the North Karnataka region of India for the first time, investigated the genetic profile of neuroligin 3 gene in the Indian autistic population by DNA sequencing, and reported novel molecular-level changes.

## Methods

### Subjects

In the North Karnataka region of India, 1870 mentally ill children below 18 years of age were diagnosed using the Diagnostic and Statistical Manual of Mental Disorders (https://www.psychiatry.org/psychiatrists/practice/dsm) and the International Classification of Diseases, 10th revision (https://www.who.int/classifications/icd/icdonlineversions/en/). From these children, 150 autistic children were identified, of whom 108 children were included in this study (n_male_ = 85, n_female_ = 23; age range, 5‒18 years, and mean age, 11.7 ± 3.5 years). Children with associated medical conditions, including fragile X syndrome, chromosomal abnormalities, and metabolic disorders were excluded from the study. Furthermore, 150 age-, sex-, and ethnicity-matched healthy control children were also included in the study (n_male_ = 100, n_female_ = 50; age range, 5‒18 years, and mean age, 11.0 ± 2.0 years; p = 0.04). Shri B.M Patil Medical College, Hospital and Research Centre, BLDE (Deemed to be University), Vijayapura (Ref No: BLDE (DU) IEC/337-2018-19) approved the study. Clinical samples were obtained after receiving informed consent from the parents/guardian of the respective child.

### DNA sequencing

Genomic DNA was extracted from peripheral blood using a DNA extraction kit (DNeasy Blood and Tissue Kit, Qiagen, Hilden, Germany) as per the manufacturer’s instructions. All seven exonic regions of the NLGN3 gene were amplified by polymerase chain reaction (PCR) using PCR reaction kits (New England Biolabs, Ipswich, MA, USA) with specifically designed primers. Sequencing of the amplified product was performed on an ABI 3500 DNA analyzer using a Big Dye terminator version 3.1 cycle sequencing kit (Applied Biosystems, Waltham, MA, USA). Sequencing results were analyzed on DNA Sequencing Analysis Software v5.4 (Applied Biosystems).

### Bioinformatics analysis

### The pathogenic effects of non-synonymous variants were analyzed using the following tools:

‒ PROVEAN (Protein Variation Effect Analyser, http://provean.jcvi.org/seq_submit.php), which predicts the impact of amino acid substitutions on the biological function of a protein.

‒ PolyPhen-2 (Polymorphism Phenotypic-2, http://genetics.bwh.harvard.edu/pph2/index.shtml), which is a tool that predicts the impact of amino acid substitutions on the structure and function of human proteins using straightforward physical comparative considerations.

‒ PHD SNP (https://snps.biofold.org/phd-snp/phd-snp.html), a predictor of human deleterious SNPs.

‒ SNP & GO (https://snps.biofold.org/snps-and-go/snps-and-go.html), which predicts disease-associated variants using Gene Ontology terms.

‒ PANTHER (Protein Analysis through Evolutionary Relationships; http://www.pantherdb.org/), which uses evolutionary relationships to infer gene function.

‒ SNAP2 (https://www.rostlab.org/services/snap/), which predicts the functional effects of sequence variants.

The evolutionary conservation of the missense variant was investigated by Clustal Omega (https://www.ebi.ac.uk/Tools/msa/clustalo/) using *NLGN3* sequences from different species. Homology modelling of wild-type and mutant NLGN3 proteins was conducted using a fully automated protein structure homology-modelling server (Swiss Model; https://swissmodel.expasy.org/), and the results were visualized and analyzed using the UCSF Chimera program.

The Uniprot accession numbers were Q9NZ94 (*Homo sapiens*), Q8BYM5 (*Mus musculus*), Q62889 (*Rattus*), F1Q3I9 (*Canis lupus*), G3MXP5 (*Bos taurus*), A0A2I2UDX2 (*Felis catus*), E9KFA0 (*Gallus gallus*), G3RBW3 (*Gorilla gorilla*), and G7NRV3 (*Macaca mulatta*).

### Statistical analysis

The obtained data were tabulated and analyzed via SPSS version 15.0 (SPSS Inc., Chicago, IL, USA). Quantitative statistical analysis was performed using the two-tailed Student t-test. Data are presented as mean ± SD. The chi‐square test was used to calculate the genotype frequencies of cases and controls. The frequency of the variant was estimated using the Hardy-Weinberg equilibrium (HWE). The risk association between the novel missense mutation and autism was calculated by analyzing odds ratios along with their 95% CIs through allelic, dominant, and recessive genetic models by logistic regression. A p-value < 0.05 was considered to indicate statistical significance.

## Results

**Fig. 1.**
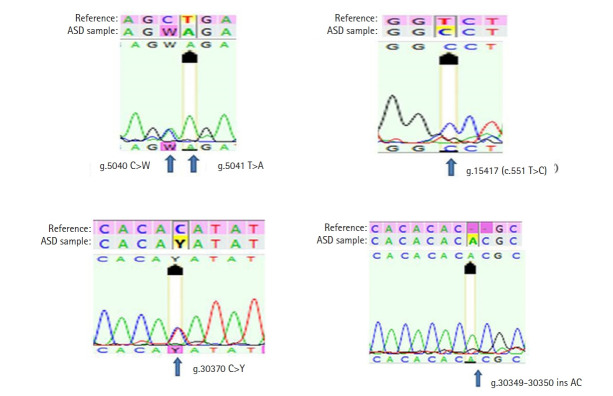
All the four variants of the *NLGN3* gene recorded in the autistic population. ASD, autism spectrum disorder.

In the present study, all seven exonic regions of the *NLGN3* gene were analyzed in 108 individuals. One coding sequence variant and four non-coding sequence variants (two 5'-untranslated region [UTR] variants and two 3'-UTR variants) were observed. The coding sequence variant c.551 T>C (p.V184A) was a missense variant observed in 27 autistic children (25%). The 5'-UTR variant g.5040 C>W was found in one (0.92%) and g.5041 T>A was found in five (4.6%) autistic children, while the 3'-UTR variant g.30370 C>Y was observed in 75 (69.4%) and g.30349‒30350 Ins AC was observed in 21 (19.4%) autistic children. The g.5040 C>W and g.30370 C>Y variants were heterozygous (Table 1, Fig. 1). All the variants are novel and have not been reported previously in any of the in-house human SNP databases such as dbSNP, 1000genomes, ExAc, ClinVar, HapMap, and gnomAD. The clinical features of all the 27 autistic children in whom the novel missense mutation p.V184A was recorded are summarised in Table 2.

**Fig. 2.**
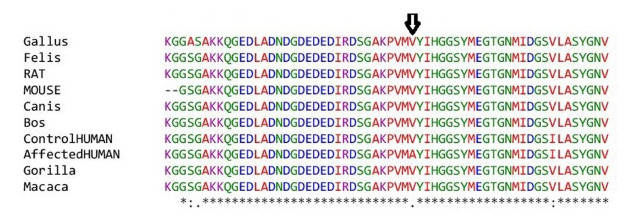
Multiple sequence alignment of neuroligin 3 protein sequences. The amino acid residue at 184 is arrowed.

**Fig. 3.**
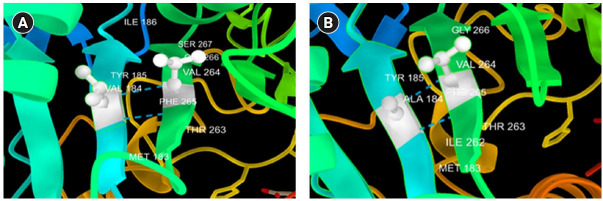
3D structure of V184A substitution using Chimera software. The wild-type residue (A) and the mutant residue (B).

**Fig. 4.**
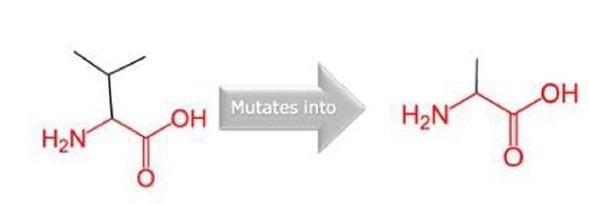
Amino acid structure of valine to alanine.

The novel missense variant c.551 T>C (p.V184A) was predicted to have a deleterious effect on the function of the NLGN3 protein by PolyPhen2, PROVEAN, PANTHER, SNP&GO, PHD-SNP, and SNAP2 (Table 3). Multiple sequence alignment of NLGN3 was carried out using Clustal Omega, which indicated that the valine at residue 184 is fully conserved across different species ranging from chickens to humans (Fig. 2). Three-dimensional models for the wild-type NLGN3 protein and the mutated NLGN3 (p.184A) protein were generated using the homology-modelling server Swiss Model. The wild-type protein had a Q_mean_ of ‒1.24 and the mutated NLGN3 protein had a Q_mean_ of ‒1.32. At residue V184, two H-bonds were observed with V264 in both wild-type (V184) and mutant (A184) proteins (Fig. 3 and 4), and the mutant residue was smaller than the wild-type residue, which might lead to a loss of interactions with other molecules.

Basic information containing allelic frequency, minor allele frequency distribution, HWE p-value, odds ratios (ORs), and 95% CIs of c.551T>C is shown in Table 4. c.551T>C was in HWE in the control group (p > 0.05). The frequency of the C allele of c.551T>C was significantly higher in cases than in controls (2.5% vs. 1%) which suggests that the C allele of the novel frameshift mutation c.551T>C was associated with an increased risk of autism (OR, 24.7; 95% CI, 5.7 to 106.4; p = 0.001) (relative risk, 18.9; 95% CI, 4.5 to 77.2; p = 0.002). Furthermore, we assumed that the minor allele of c.551T>C was a risk factor compared with the wild-type allele. Three genetic models (codominant, dominant, and recessive) were applied to analyze the significance of the association between the novel mutation and autism risk using logistic regression. A statistically significant increase in the risk of autism was observed for the C/C genotype compared to the wild-type T/T genotype (OR, 96.68; 95% CI, 5.8 to 1,607.1; p = 0.001), which corresponds to a 96.68-fold increased risk of autism under the codominant model. The combined genotype (T/C + C/C) also significantly increased the autism risk under the dominant model (OR, 24.7; 95% CI, 5.7 to 106.4; p = 0.001) (Table 5). We implemented a stratification analysis by sex to evaluate the sex association between the novel mutation allele and autism. We found that the C/C genotype was associated with an increased risk for autism in males (OR, 55.1; 95% CI, 3.3 to 929.7; p = 0.005) under the codominant model, as was the combined genotype (T/C + C/C) under the dominant model (OR, 26.6; 95% CI, 3.5 to 204.1; p = 0.002). The C/C genotype was also associated with an increased risk for autism in females (OR, 55.4; 95% CI, 3.0 to 1,015.2; p = 0.006) under the codominant model, as was the combined genotype (T/C + C/C) under the dominant model (OR, 31.5; 95% CI, 3.7 to 270.3; p = 0.001) under the dominant model (Table 6).

## Discussion

Autism (MIM 209850) is a heterogeneous neurological disorder manifesting before the age of three [1,3]. Autism results in impaired social interactions, impaired communication, and abnormal behavior [1,13]. Genetic research on autism has made great progress in the past few years. Neuroligin, a postsynaptic transmembrane protein involved in synaptogenesis, has been predicted to be a promising candidate gene for autism and other neurological disorders [15-18]. Neuroligin 3 shows diverse genetic alterations in autism. In India, autism is still understudied, and insufficient experimental data are available on its clinical aspects. To our knowledge, this is the first study from India on the role of the *NLGN3* gene in autism. The first-ever study to show the role of *NLGN3* in autism recorded a missense variant (R451C) that alters the binding of neuroligin to neurexin, resulting in the abnormal formation, stabilization, and recognition of specific synapses essential for communication process that are defective in autism, was conducted in 2003 [9]. Later studies showed that neuroligin gene mutations were implicated in rare cases of autism. Despite the rarity of its involvement, the components involved in the synaptogenesis and synaptic structures remain excellent functional candidates for future molecular genetic studies of autism and related disorders [19]. Subsequent studies reported different mutations in different regions of the *NLGN3* gene across the world. Mutated versions of the NLGN3 protein with the missense variants Pro514Ser and Arg597Trp do not reach the plasma membrane in the cell, preventing it from interacting with the neurexin protein in the human brain [20]. Certain missense variants may influence males’ susceptibility to ASD [21]. Hence, *NLGN3* may be a candidate gene for the male predominance of autism. Several missense variants in *NLGN3* account for non-syndromic forms of intellectual disability associated with autism [20]. Moreover, missense variants with pathogenic effects are possible etiological factors for autism [22]. Coding sequence variations in *NLGN3* and *NLGN4* are rare, but contribute to the aetiology of autism [23]. In addition to missense variants, but intronic and non-synonymous variants also affect the regulatory region such as enhancers and promoters associated with histone modification sites (nuclease-accessible sites and transcription factor binding sites), making modest contributions to the pathogenesis of ASD [23-25]. Other forms of genetic alterations, including splice variants and non-coding sequence variants, may also lead to the potentially abnormal function of neuroligin in autism [26,27].

All previous studies strongly suggest that neuroligin plays a heterogeneous role in the aetiology of autism. No molecular study of neuroligin on autism in India has been conducted to date. We studied 108 autistic individuals and found the novel missense variant c.551T>C in the *NLGN3* gene, which was not recorded previously in any autistic population around the globe. In our study, the C/C genotype of c.551T>C was significantly more common in the autistic children than in the controls (p = 0.001). In particular, there was a significantly 96.68-fold increased risk of autism (p = 0.001) associated with this genotype. However, the combined T/C+C/C genotype showed a significantly 24.7-fold increased risk of autism (p = 0.001), implying that the T allele may be a protective factor and people who carry this allele may be less likely to develop autism. In both male and female children, the C/C genotype of c.551T>C showed a significantly similar risk ratio (55.1-fold vs. 55.4-fold). The c.551T>C variant was predicted to be a pathogenic variant of NLGN3 by the PROVEAN, PolyPhen2, PANTHER, PHD-SNP, SNPs & GO, and SNAP2 prediction tools. The V184 residue constitutes a highly conserved amino acid that shows a strong evolutionary relationship over different species. A structural analysis of the wild-type and mutant protein showed variation in structural integrity and a reduction of the stability of the mutant protein relative to the wild-type; the mutant residue was smaller than the wild-type residue. This might lead to a loss of interactions with other molecules because it is located in the extracellular region of the NLGN3 protein. However, as a limitation of this study, the identification of a coding sequence variant is not sufficient to disclose the exact role of the gene in a disease that has a complex genetic architecture. Moreover, we also recorded four novel non-coding sequence variants (g.5040 C>W, g.5041 T>A, g.30349-30350 Ins AC, and g.30370 C>Y). These non-coding variants may also play a role in posttranslational modification. Our study is only preliminary basic research, and further functional analysis of novel mutations in the neuroligin pathway will provide a better understanding of the involvement of the *NLGN3* gene in autism in the Indian population.

The findings of our study suggest that the novel missense variant c.551T>C (p.V184A) causes abnormalities in the NLGN 3 protein, which may lead to deficits in synaptogenesis, in the Indian autistic population. Neuroligin is probably a candidate gene for future molecular investigation and functional analyses in the Indian autistic population.

## Figures and Tables

**Table 1.** List of variants and frequency of variants in our study population

Gene	Mutation type	Nucleotide change	Amino acid change	Exon	Frequency of mutation in autism group (%)	Frequency of mutation in control group (%)
*NLGN3*	5′ UTR	g.5040 C>W	-	1	1 (0.92)	0
	5′ UTR	g.5041 T>A	-	1	5 (4.6)	0
	Missense	g.15417 [c.551 T>C]	p.V184A	4	27 (25)	2 (1.3)
	3′ UTR	g.30349-30350 Ins AC	-	7	21 (19.4)	1 (0.7)
	3′ UTR	g.30370 C>Y	-	7	75 (69.4)	6 (4)

Autistic individuals (n = 108), n_male_ = 85, n_female_= 23; age range, 5 to 18 years, and mean age, 11.7 ± 3.5, p = 0.03. Furthermore, 150 age-, sex-, and ethnicity-matched control children were also included in the study (n_male_ = 100, n_female_ = 50; age range, 5 to 18 years, and mean age, 11.0 ± 2.0, p = 0.04). The NG_015874.1 reference sequence was used for genomic DNA variant nomenclature, the ENST00000374051.7 reference sequence was used for coding region variant nomenclature, and the ENSP00000363163.3 reference sequence was used for protein variant nomenclature. The nomenclature followed the Human Genome Variation Society guidelines. UTR, untranslated region.

**Table 2.** Clinical features of autistic children with the novel missense mutation c.551T>C in the *NLGN3* gene

No.	Sex/ Age (y)	CARS score and severity	IQ	Age of mother^a^	Age of father^b^	Prenatal damage	Postnatal damage	Consanguineous marriage	Co-morbid condition
Child 1	M/13	Severe, 51	20	32	36	None	Birth asphyxia	Yes	None
Child 2	F/10	Severe, 50	25	25	30	Preeclampsia	Respiratory illness	Yes	None
Child 3	M/14	Severe, 46	30	32	35	Infection during Pregnancy	None	No	None
Child 4	M/11	Severe, 48.5	30	26	32	Hyperthyroidism	None	Yes	None
Child 5	F/14	Severe, 47.5	20	28	33	None	Birth asphyxia	Yes	None
Child 6	M/10	Severe, 52	30	32	37	None	Delayed crying	Yes	None
Child 7	M/9	Severe, 43.5	20	34	43	None	None	No	None
Child 8	F/8	Severe, 49.5	30	16	36	Preeclampsia	None	No	None
Child 9	M/10	Mildly moderate, 37	35	22	30	None		Yes	None
							None		
Child 10	M/13	Severe, 51.5	30	33	38	Maternal hypertension	None	Yes	None
Child 11	M/11	Severe, 42	20	22	30	None	Delayed crying	Yes	None
Child 12	F/13	Severe, 40.5	20	28	35	None	None	No	None
Child 13	F/11	Mildly moderate, 35	65	32	38	infection during pregnancy	Birth asphyxia	No	None
Child 14	M/10	Mildly moderate, 30	65	25	32	Maternal hypertension	None	No	None
Child 15	M/12	Mildly moderate, 36	35	28	36	None	Delayed crying	No	None
Child 16	M/14	Severe, 50	20	20	29	Maternal hypertension	None	Yes	None
Child 17	M/9	Mildly moderate, 32	20	33	39	Preeclampsia	None	No	None
Child 18	F/8	Severe, 50	20	30	36	Hyperthyroidism	Respiratory illness	No	None
Child 19	M/10	Mildly moderate, 33	25	26	30	None	None	Yes	None
Child 20	M/11	Mildly moderate, 36	40	19	29	None	None	Yes	None
Child 21	M/8	Mildly moderate, 35	35	28	34	None	Feeding problem	Yes	None
Child 22	F/10	Severe, 56	20	30	36	Infection during pregnancy	Respiratory illness	No	None
Child 23	F/13	Severe, 49	25	35	45	None	Delayed crying	Yes	None
Child 24	M/12	Mildly moderate, 33	30	18	25	Maternal hypertension	Feeding problem	No	None
Child 25	M/9	Severe, 53	30	29	38	None	None	No	None
Child 26	M/11	Mildly moderate, 32	20	36	43	None	None	Yes	None
Child 27	F/12	Mildly moderate, 35	30	35	38	Preeclampsia	None	Yes	None

CARS, Childhood Autism Rating Scale.

a Age of mother at childbirth.

b Age of father at childbirth.

**Table 3.** Pathogenicity of the missense variant as identified by five *in silico* tools

PROVEAN	PHD-SNP	SNP & GO	SNAP2	Polyphen-2	PANTHER
Deleterious	Disease	Disease	Effect	Probably damaging	Probably damaging
score: ‒3.094	reliability index: 8	probability: 0.682	score: 23	score: 0.982	

*NLGN3* mutation: missense, nucleotide change: c.551 T>C, amino acid change: p.V184A. PROVEAN: “deleterious” if the prediction score is </-2.5, “neutral” if the prediction score is >/- 2.5. SNP & GO: If the probability is >0.5, then it is predicted to be a disease-causing nsSNP. SNAP2: “neutral” if the score is from 0 to ‒100, “effect” if the score is from 0 to 100. PolyPhen-2: “probably damaging” indicates the strongest disease-causing ability with a score close to 1; “possibly damaging” refers to less disease-causing ability with a score of 0.5‒0.8; and “benign” corresponds to no alteration of protein function, with a score closer to 0. PHD-SNP: if the probability is >0.5, the mutation is predicted as “disease” and if it is less than <0.5, the mutation is predicted to be “neutral.”

**Table 4.** Basic information on the novel missense mutation c.551T>C and MAF between cases and controls

Variation type	Position	Minor allele	MAF		HWE-p	Odds ratio (95% CI)	p-value	Relative risk (95% CI)	p-value
			Case	Control					
Missense	c.551	C	0.25	0.01	0.99	24.7 (5.7‒106.4)	0.001*	18.9 (4.5‒77.2)	0.002*

HWE p-values were calculated using the 2-sided chi-square test. MAF, minor allele frequency; HWE, Hardy-Weinberg equilibrium; CI, confidence interval.

* p < 0.05 indicates statistical significant.

**Table 5.** Association between the novel missense mutation c.551T>C and the risk of autism in genetic models

Models	Genotype	No. (%)		OR (95% CI)	p-value
		Case	Control		
Codominant	T/T	81	148	1 (ref.)	-
	T/C	1	2	0.69 (0.1‒7.7)	0.38
	C/C	26	0	96.68	0.001*
				(5.8‒1,607.1)	
Dominant	T/T	81	148	1 (ref.)	-
	T/C-C/C	27	2	24.7	0.001*
				(5.7‒106.4)	
Recessive	T/T-T/C	81	150	1 (ref.)	-
	C/C	26	0	96.68	0.001*
				(5.8‒1,607.1)	

OR, odds ratio; CI, confidence interval.

* p < 0.05 indicates statistical significant.

**Table 6.** Analysis of novel missense mutation c.515T>C genotype and autism risk in males and females based on logistic tests

Model	Genotype	Male		OR (95% CI)	p-value	Female		OR (95% CI)	p-value
		Case	Control			Case	Control		
Codominant	T/T	76	99	1 (ref.)	-	14	49	1 (ref.)	---
	T/C	0	1	0.39	0.56	1	1	2.2	0.57
				(0.02‒9.6)				(0.13-37.2)	
	C/C	18	0	55.1	0.005*	8	0	55.4	0.006*
				(3.3‒929.7)				(3.0-1015.2)	
Dominant	T/T	67	99	1 (ref.)	-	14	49	1 (ref.)	---
	T/C-C/C	18	1	26.6	0.002*	9	1	31.5	0.001*
				(3.5‒204.1)				(3.7-270.3)	
Recessive	T/T-T/C	67	100	1 (ref.)	-	15	50	1 (ref.)	---
	C/C	18	0	55.1	0.005*	8	0	55.4	0.006*
				(3.3‒929.7)				(3.0-1015.2)	

OR, odds ratio; CI, confidence interval.

* p < 0.05 indicates statistical significant.
